# Syrian hamster as an ideal animal model for evaluation of cancer immunotherapy

**DOI:** 10.3389/fimmu.2023.1126969

**Published:** 2023-02-27

**Authors:** Yangyang Jia, Yanru Wang, Louisa S Chard Dunmall, Nicholas R. Lemoine, Pengju Wang, Yaohe Wang

**Affiliations:** ^1^ State Key Laboratory of Esophageal Cancer Prevention & Treatment, School of Basic Medical Sciences, Academy of Medical Sciences, Zhengzhou University, Zhengzhou, China; ^2^ Centre for Cancer Biomarkers & Biotherapeutics, Barts Cancer Institute, Queen Mary University of London, London, United Kingdom

**Keywords:** Syrian hamster, tumor model, cancer immunotherapy, immune checkpoint inhibitor, cytokine, adoptive cell therapy, cancer vaccine, oncolytic virus

## Abstract

Cancer immunotherapy (CIT) has emerged as an exciting new pillar of cancer treatment. Although benefits have been achieved in individual patients, the overall response rate is still not satisfactory. To address this, an ideal preclinical animal model for evaluating CIT is urgently needed. Syrian hamsters present similar features to humans with regard to their anatomy, physiology, and pathology. Notably, the histological features and pathological progression of tumors and the complexity of the tumor microenvironment are equivalent to the human scenario. This article reviews the current tumor models in Syrian hamster and the latest progress in their application to development of tumor treatments including immune checkpoint inhibitors, cytokines, adoptive cell therapy, cancer vaccines, and oncolytic viruses. This progress strongly advocates Syrian hamster as an ideal animal model for development and assessment of CIT for human cancer treatments. Additionally, the challenges of the Syrian hamster as an animal model for CIT are also discussed.

## 1 Introduction

With increasing incidence and mortality, cancer is a major public health problem worldwide and is a major obstacle to increasing life expectancy (1, 2). Cancer immunotherapy (CIT) works by stimulating or re-invigorating the body’s immune system to directly attack cancer cells (3). In recent years, the number of approved CIT drugs has increased dramatically and it has become a mainstay cancer therapy alongside traditional therapeutic options. Despite the widespread application of CIT in human cancers, only a minority of patients benefit from these therapies (4). Therefore, there is an urgent need for an ideal animal model that can accurately reflect the complexity of the immunosuppressive human tumor microenvironment to predict the immune response and efficacy of immunotherapy, promoting the development of CIT and reducing the failure rate of clinical trials.

Syrian hamster (Mesocricetus auratus) belongs to the Cricitinae family of hamsters and is commonly known as the golden hamster. These animals originated in Syria and are naturally found in the arid, temperate southeast Europe and Asia Minor (5). Syrian hamsters currently used as laboratory animals originated from a litter captured in 1930 (5, 6). There is mounting evidence that the Syrian hamster is highly similar to humans in anatomy, physiology, and pathology (7–9). Their similarity is especially valuable in terms of the similarity of pathophysiology, *i.e* the occurrence, development, symptoms, pathology, and outcomes of disease (10). Syrian hamsters have a short reproductive cycle, are docile, and are easy to raise and handle (8, 11).

It has been reported that the human granulocyte-macrophage colony-stimulating factor (GM-CSF) is immunologically functional in hamsters (12) and recently we reported that human interleukin (IL)-12 was biologically active towards the immune cells of Syrian hamsters (13). The human cytokines IL-21 and IL-2 have also demonstrated immunological activity in hamsters (14, 15). The Syrian hamster macrophage migration inhibitory factor (MIF) is structurally and functionally similar to humans and significantly enhanced tumor growth and promoted tumor-associated angiogenesis in Syrian hamster tumor models (16). An animal model with an intact and functional immune system is indispensable for the development and characterization of cancer immunotherapy efficacy and potential toxicities, therefore, the Syrian hamster is the preferred animal model in many fields of medical research. In this review, we discuss the current landscape of the Syrian hamster tumor models, the promises and challenges of the Syrian hamster as a model for evaluating cancer immunotherapy.

## 2 Syrian hamster tumor models

### 2.1 Syngeneic tumor models

**Figure 1:**
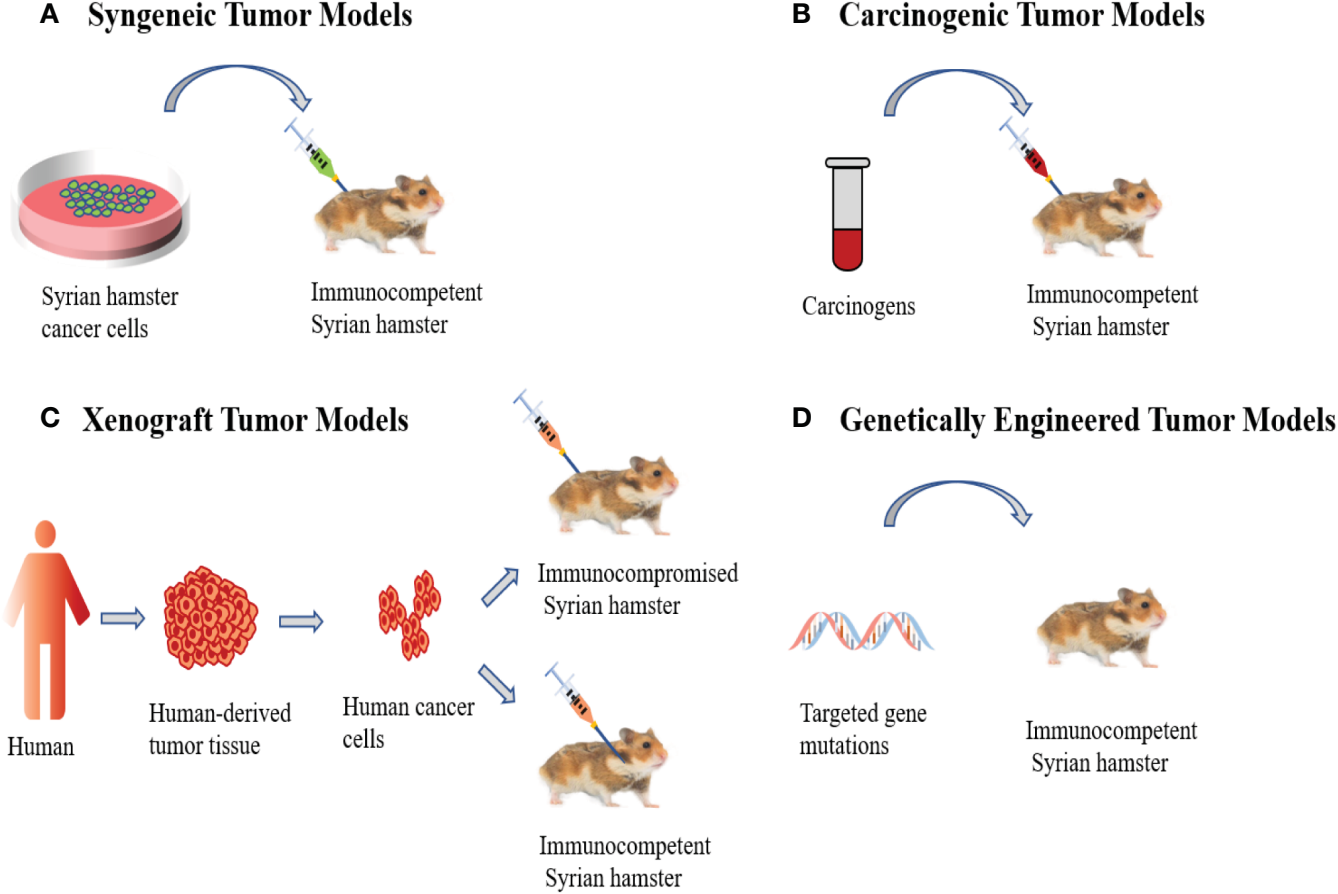
Syrian Hamster Tumor Models. **(A)** Syngeneic tumor models involve the transplantation of *in vitro* cultured Syrian hamster cancer cells into immunocompetent Syrian hamsters. **(B)** Carcinogenic tumor models are induced by carcinogens, which allows tumors to undergo *de novo* formation. **(C)** Human xenograft tumor models utilize human cancer cells to inject into immunocompromised Syrian hamsters or the cheek pouches of immunocompetent Syrian hamsters. **(D)** Genetically engineered models are the application of genetic engineering techniques to modify hamster genes to induce cancer development.

Syngeneic tumor models are the most used preclinical models for evaluating cancer immunotherapy and refer to the transplantation of *in vitro* cultured tumor cell lines into immunocompetent animals (**Figure 1A**), typically including subcutaneous, intraperitoneal disseminated and orthotopic tumor models. In recent years, Syrian hamsters have been widely used in preclinical research as tumor models. The Syrian hamster model can better simulate the development of human cancer and reproduce the genomic heterogeneity of human cancer and the complexity of its microenvironment (8, 17).

It has been reported that the hamster peritoneal omentum-associated lymphoid tissue is anatomically equivalent to the human milky spots, which are mainly composed of macrophages and are closely related to the spread of cancer cells (18, 19) The Syrian hamster cheek pouch lacks an intact lymphatic drainage pathway and is considered immune privileged, which supports the long-term survival of transplanted foreign tissue without immunological rejection (5). It was previously reported that human myofibrosarcoma (MFS-l) and melanoticmelanoma (ME-l) transplanted into the cheek pouch of Syrian hamsters were fully sensitive to colchicine stimulation throughout growth and that this sensitivity persisted after multiple consecutive transplants, thus providing support for the hamster-human tumor system as a tool to evaluate human antitumor agents (20). The Syrian hamster pancreatic cancer cell line HaP-T1 has been established and exhibits tumorigenicity on syngeneic Syrian hamsters, and histologically showed obvious epithelial characteristics similar to human pancreatic cancer. Further studies have shown that hamster N-terminal Sonic hedgehog (SHH) is 99% identical to human, while N-SHH promotes desmoplasia in human pancreatic cancer (21, 22). Researchers have also established the Syrian hamster pancreatic carcinoma cell line SHPC6, which when injected into peritoneal cavity, forms disseminated tumor nodules and nodules attached to the pancreas, faithfully simulating patients with advanced pancreatic cancer metastasis (23). Additionally, the syngeneic Syrian hamster glioma orthotopic model showed hypercellularity and necrotic areas, consistent with the characteristics of human glioma (24). At present, the Syrian hamster syngeneic tumor models used for research include pancreatic cancer (25–27), oral cancer (28), gallbladder cancer (29), glioma (24), kidney cancer (23, 30), liver cancer (23), lung cancer (23) and breast cancer (31). However, the challenge with all syngeneic tumor models is that they cannot reflect the multi-stage process of carcinogenesis due to the direct engraftment of malignant cells, therefore, these models are not suitable for the evaluation of the application of anti-tumor immunotherapy in the early stages of tumorigenesis (32).

### 2.2 Carcinogenic tumor models

Oncogenic tumor models are primarily caused by carcinogens (**Figure 1B**). The instability of the genome allows tumors to undergo *de novo* formation in the microenvironment. This produces great genomic complexity and accurately reflects the occurrence and development of tumors in humans (33). It has been reported that N-nitroso-bis(2-oxopropyl) amine (BOP) can induce pancreatic ductal adenocarcinoma in Syrian hamsters and its histology, morphology, biology, pathology, and genetics are similar to humans. For example, its development from ductal cells, local invasion, distant metastasis, cachexia, and point mutations in the *K-ras* gene (34–37). In addition, studies have shown that the Syrian hamster cholangiocarcinoma model induced by the combination of Clonorchis Sinensis infection and dimethyl nitrosamine resulted in chronic inflammation, bile duct injury and atypical bile duct hyperplasia, with progression to cholangiocarcinoma, histopathologically similar to human liver fluke infection-associated cholangiocarcinoma (38–40). The chemically induced Syrian hamster cheek pouch carcinogenesis model can accurately reflect a series of common processes seen in human oral precancerous lesions and carcinogenesis, including mutations and expression changes of oncogenes and tumor suppressor genes. Histologically, the cells showed varying degrees of atypia and differentiation, similar to those seen in human squamous cell carcinoma (41–46). Researchers used 9,10-dimethyl-1,2-benzthracene (DMBA) to induce the formation of cheek pocket squamous cell carcinoma in Syrian hamsters and established the Syrian hamster cheek pocket squamous cell carcinoma cell line. Subsequently, the cell lines were transplanted into the subcutaneous and cheek pouch of hamsters and treated with the oncolytic adenovirus HAdV5 exhibiting significant antitumor effects (28). In addition, it has been reported that chemical carcinogens could induce lung cancer in Syrian hamsters (47–52), and the N-nitrosobis (2-oxopropyl) amine (BOP) induced Syrian hamster lung cancer model faithfully mimics human small cell and non-small cell lung cancer (NSCLC) (47, 51, 52). Histopathologically, it shows a subtype of adenocarcinoma, which is very similar to the type of adenocarcinoma of human non-small cell lung cancer (NSCLC), and the common feature with human lung cancer is that tumor tissues overexpress cyclooxygenase-2 (COX-2) and nuclear factor kappa B (NF-kappaB), which are important factors in the pathogenesis of lung cancer (44). Compared with mice and rats, the streptozotocin-induced Syrian hamster hepatocellular carcinoma tumor model has the advantage of high incidence and short latency, making it an ideal animal model for assessing hepatocarcinogenesis (53). Researchers have also used N-methyl-N-nitrosourea (MNU) to induce hamster breast cancer, and histopathological analysis showed that this animal model resembled human high-grade poorly differentiated breast adenocarcinoma. At the same time, they established the first immortalization of a Syrian hamster breast cancer cell line HMAM5, with a high tumorigenic rate in female hamsters (31). Additional carcinogen-induced cancers in Syrian hamsters include kidney cancer (51, 54, 55), cholangiocellular tumors (52), endometrial adenocarcinoma (56) and laryngeal cancer (57).

### 2.3 Xenograft tumor models

Human xenograft tumor models are established by transplantation of human-derived tumor cell lines or tumor tissues into immunocompromised Syrian hamsters (**Figure 1C**). Recently, we created a new pancreatic cancer xenograft model using *IL2RG* gene knockout Syrian hamsters (named ZZU001) with IL-2, IL-4, IL-7, IL-9, IL-15, and IL- 21 receptor function deficiencies, resulting in a significantly decrease in T lymphocyte activity and non-functional B and NK cells, producing a severely immunocompromised state (8, 11). Subcutaneous and orthotopic transplantation of a panel of human pancreatic cancer (PC) cell lines into ZZU001 Syrian hamsters and immunodeficient mice demonstrated that only in ZZU001 did multi-organ metastases similar to those seen in human pancreatic cancer develop. PC tissue from Syrian hamsters exhibited desmoplastic reactions in both stromal and epithelial-to-mesenchymal transition phenotypes, whereas PC tissues from immunodeficient mice did not show this feature. In addition, we transplanted an established human esophageal cancer (EC) cell line (SBRC-EC01) into immunodeficient ZZU001 Syrian hamsters, resulting in a subcutaneous tumor with a pathological structure similar to the primary tumor, but SBRC-EC01 has no tumorigenicity in B-NDG mice and BALB/c nude mice. This suggests the Syrian hamster is a very valuable animal model for studying treatment responses in human PC and EC (8).

Most commonly, human tumor models are established by subcutaneous inoculation of human tumor cells into immunodeficient mice, however, in these models, the histological features of the primary tumor are not reflected (17) The cheek pouches of hamsters possess immune-privileged sites that allow human tumor transplantation to form a distinctive xenograft tumor model. Sakakibara et al. (58) showed that when human malignant tumor tissue-derived cell lines were transplanted into the cheek pouches of Syrian hamsters, the histology of these tumors was consistent with the primary tumor. This was not reflected when the same model was established in mice (59). In addition, serial transplantation of human lymphomas in neonatal hamsters has also been reported, a model that may be a practical system for the study of immunotherapeutic agents, however, no similar reports have been reported in neonatal mice (60, 61). The establishment of human osteosarcoma model in newborn Syrian hamsters has also been reported (62). In conclusion, although xenograft mouse models have become common models for evaluating tumor progression and the efficacy of cancer drugs, they do not faithfully reflect human tumor progression. However, the immune-deficient Syrian hamster model ZZU001 can be used to better simulate human tumors and show the histological and pathological characteristics of human tumors.

### 2.4 Genetically engineered tumor models

Genetically engineered models involve the application of genetic engineering techniques to modify hamster genes to induce cancer development (**Figure 1D**). Currently, hamster transgenic models mainly include *KCNQ1* knockout, *TP53* knockout and *IL2RG* knockout as discussed above (8, 63, 64). *KCNQ1* is a gene encoding a potassium channel that is widely expressed in human and rodent tissues, and *KCNQ1* functions as a tumor suppressor gene in the gastrointestinal tract of both humans and mice (65). Li et al. created the first genetically engineered *KCNQ1* knockout hamster cancer model by CRISPR/Cas9 genetic engineering technology, and the homozygous *KCNQ1* knockout hamsters showed severe physical discomfort response at day 70. 85.7% of the hamsters had visible cancers at necropsy, including T-cell lymphoma, plasma cell tumors, hemangiosarcomas, and suspected myeloid leukemia, and developed multi-organ infiltrates (63). In addition, Miao et al. used CRISPR/Cas9 genetic engineering technology to generate *TP53* knockout hamsters. In *TP53* homozygous mutant hamsters, lymphomas, hemangiosarcomas, and myeloid leukemias were the predominant types of cancer developed, while *TP53* heterozygous hamsters mainly developed lymphomas (64). Of note, in contrast with *TP53* homozygous KO mice, hamsters developed carcinomas in several epithelial tissues that were not observed in *TP53* KO mice. *TP53*-deficient acute myeloid leukemia (AML) in human is highly malignant, has a low response rate to chemotherapy, and has a mediate overall survival of only 5-10 months (66), so there is an urgent need for effective animal models to develop new treatments. Most importantly, *TP53*-deficient hamsters can develop acute myelogenous leukemia, however, *TP53*-deficient mice generally do not develop acute myelogenous leukemia (64). Thus, hamsters can be used to evaluate new therapeutic modalities for *TP53*-deficient acute myeloid leukemia. Recently, Miao et al. established a novel *SHARPIN* knockout (KO) Syrian hamster using the CRISPR/Cas9 system, and eosinophil infiltration in esophagus and other organs was observed in S*HARPIN* KO transgenic hamsters, which may represent early symptoms of human eosinophilic esophagitis (EoE). Therefore, it is expected that *SHARPIN* KO transgenic hamsters will encapsulate the syndrome of human autoinflammatory diseases (67).

## 3 Cancer immunotherapy in the Syrian hamster model

Cancer Immunotherapy (CIT) was the breakthrough of the year 2013 (68). More than a dozen immunotherapies have been approved for cancer treatment to date, however, there are many more immunotherapies in preclinical trials and there is an urgent need for suitable preclinical models to effectively assess their suitability. CIT methods currently evaluated in Syrian hamsters include immune checkpoint inhibitors (14, 69), cytokines (13, 70–72), adoptive cell therapy (15, 60, 73), cancer vaccines (19, 74) and oncolytic viruses (14, 26, 29, 75). A summary of Syrian hamster models used for evaluation of CIT is presented in **Table 1**.

**Table 1:** The Syrian Hamster Models in Cancer Immunotherapy.

Cancer models	Cell lines	Reagents	Immunotherapy approach	Refs
Pancreatic cancer	HPD-1NR, HaK	Oncolytic adenovirus, Vaccinia virus	Oncolytic virus therapy	(9)
	HPD1NR, SHPC6, Hap-T1	Ad-TD-nsIL-12	Cytokine, Oncolytic virus therapy	(13)
	SHPC6	VVLΔTK-STCΔN1L-IL21	Cytokine, Oncolytic virus therapy	(14)
	HPD-1NR	Ad5	Oncolytic virus therapy	(76)
	PGHAM-1	Dendritic cell (DC)	Therapeutic Cancer Vaccine	(19)
	SHPC6	INGN 007	Oncolytic virus therapy	(23)
	HP-1	RGD-ΔE3-ADP-ham-IFN	Cytokine, Oncolytic virus therapy	(25)
	HaP-T1	Ad-OSM, NDV-OSM	Oncolytic virus therapy	(26)
	PGHAM-1	AdCV-hamIFN	Cytokine, Oncolytic virus therapy	(27)
	HapT1	Ad5-D24, Tumor-infiltrating lymphocytes (TILs)	Oncolytic virus therapy, Adoptive Cell Therapy	(60)
	HaP-T1	oAd/IL12/GM-RLX αPD-1	Cytokine, Oncolytic virus therapy, Immune Checkpoint Inhibitor	(69)
	Hap-T1	vvdd-tdTomato-hGMCSF	Oncolytic virus therapy, Cytokine	(70)
	HapT1	Ad5/3-E2F-d24-hIL2, rhIL-2, TIL therapy	Cytokine, Oncolytic virus therapy, Adoptive Cell Therapy	(73)
	HapT1	Ad5/3-E2F-d24-hTNF-α-IRES-hIL-2(TILT-123), Tumor-infiltrating lymphocytes (TILs)	Cytokine, Oncolytic virus therapy, Adoptive Cell Therapy	(15)
	HPD1NR	Dendritic cell (DC)	Therapeutic Cancer Vaccine	(74)
	HaK, PC1	VRX-007	Oncolytic virus therapy	(77)
	HP-1	VCN-01	Oncolytic virus therapy	(78)
	HP-1	VCN-11	Oncolytic virus therapy	(79)
	SHPC6	VRX-007	Oncolytic virus therapy	(80)
	HaP-T1, HP-1, H2T	Ad-WTLuc	Oncolytic virus therapy	(81)
	HaP-T1	AxE1CAUP	Oncolytic virus therapy	(82)
	HaP-T1, H2T	Human adenovirus	Oncolytic virus therapy	(83)
	H2T	Ad-DHscIL12	Cytokine, Oncolytic virus therapy	(84)
	HapT1	Ad5/3-E2F-d24-vIL2	Cytokine, Oncolytic virus therapy	(85)
	HapT1, HapT1 AIM2-/-	Ad5/3-d24-E2F-hTNFa-IRES-hIL2	Cytokine, Oncolytic virus therapy	(86)
	HaP-T1	Ad-IL12G Virus-loaded monocytes	Cytokine, Oncolytic virus therapy	(87)
Human Esophageal Squamous Cell Carcinoma	SBRC-EC01	Ad-TD-nsIL12	Cytokine, Oncolytic virus therapy	(17)
Glioblastoma	HamGSC-1, HamGSC-2	Delta-24-RGD	Oncolytic virus therapy	(24)
Cheek pouch SCC tumor	HPT11, HPT12	lp11w/Δ55K	Oncolytic virus therapy	(28)
Head and neck cancer	HCPC1	VVΔTKΔN1L-hIL12	Cytokine, Oncolytic virus therapy	(88)
Gallbladder cancer	G207	KIGB-5	Oncolytic virus therapy	(29, 89)
Renal cancer	HaK	KD3-IFN	Cytokine, Oncolytic virus therapy	(30)
	HaK	vvdd-tdTomato-hGMCSF	Cytokine, Oncolytic virus therapy	(70)
	HaK	VRX-007	Oncolytic virus therapy	(80, 90, 91)
	HaK	VRX-007, Ad5	Oncolytic virus therapy	(92)
	HaK	INGN 007	Oncolytic virus therapy	(93)
Lymphosarcoma	TBD 932	IFN-α	Cytokine	(71, 72)
Leiomyosarcoma	DDT1 MF-2	VRX-007	Oncolytic virus therapy	(77)
	DDT1-MF2	Ad5/3-D24-GMCSF	Cytokine, Oncolytic virus therapy	(94, 95)

### 3.1 Immune checkpoint inhibitors

Immune checkpoint inhibitors (ICI) are by far the most thoroughly studied and clinically advanced form of immunotherapy. PD-1/PD-L1 and CTLA-4 are the two most common immune checkpoint inhibitor targets and several ICI drugs have been approved by the FDA for cancer treatment. Although ICI produces more durable responses than conventional chemoradiation, response rates remain low in many cancers (3, 96), a situation that may be improved by the application of more suitable animal models during pre-clinical testing of these agents.

It has been reported that in a Syrian hamster PC subcutaneous tumor model, αPD-1 (anti-mouse) combined with oncolytic adenovirus (AdV) co-expressing IL-12, GM-CSF, and RLX (Relaxin), which can inhibit the synthesis of extracellular matrix (ECM) and degrade the overexpressed ECM in tumor tissue, to allow full dispersion of the therapeutics,exhibited stronger tumor inhibition compared with the αPD-1 group (69). In the Syrian hamster orthotopic PC model, αPD-1 combined with oncolytic AdV significantly inhibited the growth of primary and metastatic tumors without ascites formation, whereas the αPD-1 group exhibits extensive tumor metastasis and ascites formation. Further studies showed that application of oncolytic Ad/IL12/GM-RLX enhanced penetration of α-PD1 into tumor tissues, and enhanced T cell activation and infiltration into tumors (69). Both the Syrian hamster orthotopic and intraperitoneal dissemination models can faithfully mimic the characteristics of human PC and like human PC, the Syrian hamster PC models are insensitive to ICI. However, combination therapy sensitized PC to αPD-1 treatment and enhanced antitumor effects.

### 3.2 Cytokines

Cytokines were the first type of immunotherapy to be used in the clinic and have a history of over 40 years of clinical use. IFNα and IL-2 have been approved for the treatment of malignant tumors (97, 98). Cytokines are key mediators of cell-to-cell communication in the tumor microenvironment (TME) and dysregulation of cytokine expression is present in all human cancers (99). Cytokine therapy refers to the direct stimulation of immune cell growth and activity by injected cytokines. Former commonly used cytokines treatments involved mono-therapeutic use of interferons, interleukins, or granulocyte-macrophage colony-stimulating factor (GM-CSF) (3). However, studies have shown that, except for a few cancers, such as melanoma and renal cell carcinoma, single cytokine therapy in clinical trials is not satisfactory (99) and testing of multiple agents in suitable pre-clinical models is necessary for future application of cytokine therapy.

Interestingly, it appears that the similarities between hamster and human disease pathology extend to the immune system and many human cytokines are functional in the Syrian hamster. Parviainen et al. (70) reported that human GM-CSF has immunological functions in immunocompetent Syrian hamsters. In the Syrian hamster PC model, oncolytic Vaccinia virus expressing human GM-CSF can enhance specific anti-tumor immune responses by recruitment and activation of natural killer cells, monocytes, and granulocytes to enhance the anti-tumor effect. However, human GM-CSF is not functional in mice. We reported that human IL-12 stimulated the proliferation of hamster, but not murine peripheral blood mononuclear cells and induced the expression of interferon-γ and tumor necrosis factor-alpha. At the same time, we constructed an oncolytic AdV expressing non-secreting IL-12 to achieve local low-dose release of IL-12, solving the problems of short half-life and high systemic toxicity associated with IL-12. Using the Syrian hamster PC peritoneal dissemination model and orthotopic tumor models, we demonstrated a good anti-tumor effect and prolonged survival with no toxic side effects observed (13). We also demonstrated that the oncolytic Vaccinia virus (VV) expressing human IL-21 prolongs survival in an intraperitoneal dissemination hamster model of pancreatic cancer (14). Joao Manuel Santos et al. (73) demonstrated that oncolytic AdV expressing human IL-2 achieved the local release of IL-2 in hamster pancreatic cancer, improved the efficacy of adoptive cell therapy, and significantly enhanced the infiltration of CD8^+^ T cells into the tumor microenvironment, while avoiding systemic toxicity caused by a intravenous delivery of IL-2. In addition, it was reported that human IFN-α is also immunologically functional in hamsters and prolongs survival in a hamster lymphosarcoma model (71, 72). Thus, many human cytokines are biologically active in Syrian hamsters, suggesting this model as a highly suitable immunocompetent model for direct assessment of the role of human cytokines in antitumor activity. This model will also provide robust toxicity information regarding the side-effects of cytokine treatment that can only be determined in mice by use of the murine cytokine counterparts.

### 3.3 Adoptive cell therapy

Adoptive cell therapy (ACT) mainly utilizes tumor-infiltrating lymphocyte (TIL)-derived T cells or genetically engineered T cells to express tumor-recognition receptors, resulting in durable anti-tumor responses (100). TIL-based ACT relies on (i) non-myeloablative lymphoid exhaustion, (ii) tumor-specific T cells isolated from tumor tissues, expanded *in vitro*, and injected into the host, and (iii) IL-2 administration after TIL infusion (100). Currently, TIL therapy has a durable antitumor effect in melanoma patients (101), but to make TIL therapy widely available, many researchers have improved the efficacy and safety of ACT through combination therapy strategies.

Mikko Siurala et al. (60) reported for the first time that tumor-infiltrating lymphocytes (TILs) from Syrian hamsters combined with oncolytic AdV enhanced tumor cell killing ability *in vitro*, and oncolytic adenovirus enhanced the efficacy of TIL therapy. However, systemic administration of high doses of IL-2 can lead to severe systemic toxicity and even death (73). It has been reported that using ACT to treat hamster PC, the local high-dose release of IL-2 achieved by co-treatment with Ad5/3-E2F-d24-hIL2 avoided toxicity associated with systemic administration. More interestingly, Ad5/3-E2F-d24-hIL2 increased CD8^+^ T cells in the tumor microenvironment compared with systemic IL-2 administration. Therefore, Syrian hamster models have demonstrated that in ACT therapy, local release of IL-2 can effectively replace systemic administration and reduce toxicity associated with this form of therapy (73). The Syrian hamster is crucial for this modelling as they not only have immune responses that are reflective of the human scenario and support human IL-2 functions, but critically hamster models are supportive of AdV replication, a feature not shared by murine models. The first step in ACT therapy is host lymphoid depletion, but this can lead to severe cytopenia, systemic inflammatory responses, and damage to vital organs (15, 100). In order to overcome this problem, João Manuel Santos et al. (15) used a hamster PC model to demonstrate that oncolytic AdV armed with human IL-2 and human tumor necrosis factor alpha (TNF-α) could achieve antitumor effects without lymphoid depletion. Indeed, compared with lymphoid depletion, hamsters receiving oncolytic adenovirus and TILs had a significantly longer survival time and repeated AdVtreatment resulted in durable antitumor effects. The mechanism for this is postulated as dependent on AdV-induced upregulation of major histocompatibility complex (MHC) class II in draining lymph nodes, which effectively stimulates CD4^+^ T cells. These data suggest that the Syrian hamster provides a reliable preclinical animal model for ACT, accurately modeling the toxicity of treatments with human cytokines and that they are critical models for evaluating the ACT combination with oncolytic AdV therapy. Data obtained from Syrian hamster models provide more relevant and reliable evidence for the clinical translation of this therapeutic strategy.

### 3.4 Therapeutic cancer vaccines

Therapeutic cancer vaccines are designed to induce tumor regression and eradicate cancer cells by boosting a patient’s immune response and developing durable antitumor memory immune responses (102). Dendritic cells (DCs) are the most functional antigen-presenting cells (APCs) in the body and play a key role in coordinating the innate and adaptive immune responses against the tumor (103). In 2010, the first therapeutic cancer vaccine (SiPuleucel-T) was approved by the FDA, which improved overall survival for patients with refractory prostate cancer (104, 105).

To understand the potential for a DC vaccine in hamster PC, Akiyama et al. (74) reported that hamster bone marrow (BM)-derived DC (BM-DC) stimulated with hamster tumor cell lysate had an obvious anti-tumor effect on hamster subcutaneous PC. Further studies showed that tumor lysate-pulsed hamster DC could enhance activity of tumor-specific cytotoxic T lymphocytes (CTL). Akiyama et al. (19) also reported that in the hamster PC intraperitoneal dissemination tumor model, tumor lysate-treated DCs significantly inhibited the growth of orthotopic tumor cells and the formation of ascites. Further studies showed that the antitumor effect of tumor lysate-pulsed DCs was mainly due to the activation and expansion of tumor-specific CTLs. Given the lack of success in development of successful therapeutic cancer vaccines to date, the Syrian hamster tumor model should be considered for the development and testing of these regimes as it can better demonstrate the complexity of human cancer and thus has many advantages over mouse models.

### 3.5 Oncolytic virus therapy

Oncolytic viral therapy is a promising strategy for CIT. Naturally tumor-tropic viruses, or viruses genetically modified for tumor tropism are used to generate anti-tumor activity. The mechanisms by with oncolytic viruses achieve this are (i) selective replication in and killing of tumor cells, (ii) release of tumor-related antigens to induce a systemic anti-tumor immune response, and (iii) induction of potent anti-tumor immune responses *via* infiltration and activation of innate and adaptive immune cells (106). While the prospects for oncolytic viral therapy are positive, with 4 licensed treatments to date, animal models to evaluate these vectors have significant limitations. Oncolytic AdV was the first approved oncolytic virus, licensed in 2005 in China for treatment of refractory head and neck cancer. Since then, AdV has been intensively studied as an oncolytic agent as their genome and biology are well understood, safe, and easily manipulated. However, replication of AdV is species-selective and immunocompetent mouse models are not supportive of AdV replication. Therefore, the evaluation of oncolytic AdV vectors is often performed in immunodeficient mice with human xenograft tumors that do support virus replication. This limits the useful information that is produced through *in vivo* experimentation, as these models cannot adequately assess the safety of viral vectors and their role in host immune responses. Furthermore, as human AdV replicates poorly in normal and tumor tissues of mice, off target effects are difficult to determine (19, 77, 107).

The Syrian hamster is both immunocompetent and allows human AdV replication in normal and tumor tissues, and many research papers have detailed the usefulness of Syrian hamster models for investigation on oncolytic AdV (9, 13, 23–26, 28, 76–80, 82, 84–87, 90–95, 108–111). Phillips et al. (24) established the first immunocompetent Syrian hamster glioma model that is oncolytic AdV replication-permissive, which is novel platform for studying the interactions between the host immune system, tumors, and oncolytic adenoviral therapy. Further studies showed that T-cell infiltration in the tumor microenvironment was significantly increased after treatment with the oncolytic AdV Delta-24-RGD and significant prolongation of survival was achieved compared to the control group (24). Moreover, it was reported that in the Syrian hamster xenograft human esophageal tumor model, AdV-TD-nsIL12 showed strong anti-tumor effects and inhibited tumor cell proliferation and reduced microvascular density in tumor tissue (17). In addition to its advantages for testing oncolytic AdV, the immune response elicited following VV virus infection in Syrian hamsters has been shown to be partly mediated by natural killer cells in a similar manner to human infection, and this phenomenon is not seen in mice (112), suggesting the Syrian hamster as a suitable model for assessing the immune competence of the oncolytic VV (8, 9, 14, 70, 83, 88). The immune-competent Syrian hamster has also been used to evaluate oncolytic Herpes Simplex Virus (HSV) (29, 89), Reovirus (75), and Newcastle disease virus (NDV) (26), thus providing reliable preclinical studies for the development of oncolytic virus drugs. Nakano et al. (29) demonstrated that the antitumor ability of HSV G207 in athymic-free mice was significantly lower than that in immunocompetent Syrian hamsters, indicating that T-cell-mediated immune response played an important role in the local and systemic anti-tumor effects of G207. This demonstrates the necessity of using animal models with intact immune responses to evaluate oncolytic virus therapy. Interestingly, a novel therapeutic regime to eradicate established tumors by sequential use of two different oncolytic viruses was demonstrated in immunocompetent Syrian hamster PC (HPD-1NR) and kidney cancer (HaK) models (9). Sequential administration of low-dose oncolytic adenovirus and Vaccinia virus exhibited stronger antitumor effects compared to the reverse combination or single agent therapy. Further studies showed that AdV-VV treatment increased tumor-infiltrating lymphocytes and induced a higher tumor-specific immune response (9). Therefore, the immunocompetent Syrian hamster is an appropriate animal model to evaluate the combined treatment strategy of oncolytic AdV and VV as well as other oncolytic viruses.

## 4 Conclusions and prospects

The number of cancer immunotherapy drugs approved by the FDA has been increasing and cancer immunotherapy is an exciting new paradigm in cancer therapy. An ideal preclinical animal model is urgently needed to evaluate cancer immunotherapy. The US Food and Drug Administration indicates a suitable model is similar to humans in disease onset, progression, symptoms, pathology, and pathophysiology. The Syrian hamster complies with this requirement and immunocompetent hosts can accurately mimic the human scenario in terms of immune responses to disease states and pathogens. Indeed, we and others have recently reported its relevance over mouse models for modelling of SARS-CoV2 infection and disease (113). The Syrian hamster tumor model is more representative of the occurrence and development of human tumors and faithfully recapitulates the complexity of the tumor microenvironment (23, 41). Many human cytokines, including GM-CSF, IL-21, and IL-12, have immunological functions in the hamster (13, 14, 70) and this model is also favored for the examination of species-selective oncolytic viruses such as AdV, which replicate poorly in immunocompetent mice (88).

Challenges do remain with the use of the Syrian hamster model. For example, there are fewer syngeneic tumor cell lines and genetically engineered cancer models available compared with mice and a lack of specific immune reagents can hamper in-depth investigation of immune responses and cytokine changes in the TME after CIT. Moreover, there is currently no humanized Syrian hamster model. We and others are working hard to overcome these constraints, building banks of Syrian hamster tumor cell lines, transgenic hamster models, and developing research reagents to assess immune functions accurately. Thus, we believe that soon, the Syrian hamster will become a widely-used preclinical animal model that accurately simulates various human disease states. This will allow a more accelerated, refined analysis of CIT that can be confidently applied to clinical practice in a more timely manner.

## Author contributions

Conceptualization, YJ and YHW. Writing, original draft preparation, YJ and YRW. Writing, review and editing, NL, LD and YHW. Supervision, YHW and PW. Project administration, YHW. Funding acquisition, YHW and PW. All authors contributed to the article and approved the submitted version. 
